# Biomarker detection based on nanoparticle-induced ultrasonic Rayleigh scattering

**DOI:** 10.1038/s41378-024-00808-z

**Published:** 2024-12-05

**Authors:** Wangyang Zhang, Chaoshan Zhao, Haoliang Jia, Tao Liu, Jiaqian Yang, Pengfan Wu, Xiaojing Mu

**Affiliations:** Key Laboratory of Optoelectronic Technology and Systems, Ministry of Education and International Research and Development Center of Micro-Nano Systems and New Materials Technology, Chongqing University, Chongqing, 400044 China

**Keywords:** Nanoparticles, Biosensors

## Abstract

Ultrasonic biochemical detection is important for biomarker detection, drug monitoring, and medical diagnosis, as it can predict disease progression and enable effective measures to be taken in a timely manner. However, the ultrasonic technology currently used for biochemical marker detection is directly modified on the surface of the device. The associated test methods are costly and unreliable while having poor repeatability; therefore, they cannot achieve low-cost rapid testing. In this study, a detection mechanism based on the Rayleigh scattering of acoustic waves caused by nanoparticles, which causes changes in the received sound pressure, was developed for the first time. The modification of antibodies on an insertable substrate decouples the functionalization step from the sensor surface and facilitates the application of capacitive micromachined ultrasonic transducers (CMUTs) in conjunction with Au nanoparticles (AuNPs) for CA19-9 cancer antigen detection. A corresponding detection theory was established, and the relevant parameters of the theoretical formula were verified using different nanoparticles. Using our fabricated CMUT chip with a resonant frequency of 1 MHz, the concentrations and substances of the CA19-9 antigen markers were successfully measured. In the concentration range of 0.1–1000 U/mL, the receiving voltage decreased with increasing concentration. Further investigations revealed that the influence of other interfering markers in the human body can be ignored, demonstrating the feasibility and robustness of biochemical detection based on CMUTs combined with nanoparticles.

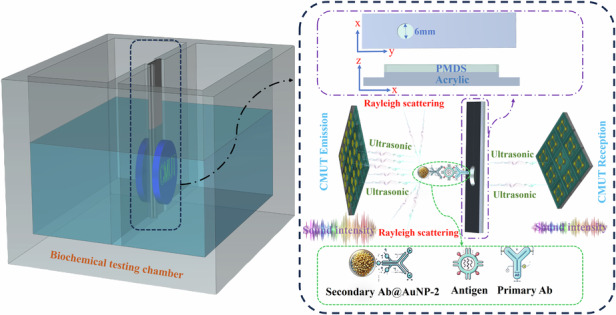

## Introduction

The ability to detect biochemical markers is important in industrial testing, environmental science, food safety, and clinical medicine, as such detection methods can be used to monitor the presence and concentrations of biochemical markers and, in some cases, prevent fatal diseases and life-threatening situations^1–4^. For example, in clinical medicine, certain biochemical markers (e.g., carbohydrate antigen 19-9 (CA19-9), cancer embryonic antigen (CEA), and alpha-fetoprotein (AFP)) are used for the early diagnosis of cancer and monitoring the effectiveness of treatment^5,6^, whereas myocardial injury markers (such as troponin and CK-MB) are used for the diagnosis of heart disease and evaluation of acute myocardial infarction^7^. In environmental science, biochemical markers of heavy metals and organic pollutants in environmental samples (such as water and soil) are used to assess and control environmental pollution, and biomarkers (such as changes in liver enzyme activity) in fish are used to assess the impact of environmental pollution on ecosystems^8^. In industrial applications, the concentrations of biochemical markers (such as glucose and lactic acid) in fermentation broth are monitored to optimize fermentation conditions and product quality. Markers such as the biochemical oxygen demand (BOD) and chemical oxygen demand (COD) in wastewater are used to evaluate wastewater treatment effects and environmental discharge compliance^9^. Various techniques have been developed for biochemical marker detection, including immunoassay methods; mass spectrometry; and optical, electrochemical, and ultrasonic detection techniques^10–12^. Compared with other methods, ultrasonic detection methods are noninvasive, portable, cost effective, and easy to perform^13^. These unique advantages make ultrasonic methods promising alternative techniques for detecting biochemical markers. Several research groups have reported the use of ultrasound to measure and detect chemical and biological components and target biomarkers of diseases, including cancer markers, gases, toxins, and pathogens^14–17^. However, this application requires repeated modifications for multiple detections, which significantly affects the sensor performance. In particular, if receptor binding is irreversible, the sensor device may not be reusable; therefore, the cost per assay will increase significantly.

Capacitive micromachined ultrasonic transducers (CMUTs) combined with nanoparticles offer a promising solution to the aforementioned problems. Currently, micromachined ultrasonic transducers are classified as CMUTs or piezoelectric micromachined ultrasonic transducers (PMUTs)^18–20^. Compared with PMUTs and their traditional counterparts, CMUTs have higher electromechanical coupling coefficients, high receiving sensitivity, and higher bandwidths^21,22^, exhibiting superior performance. AuNPs have many advantages in biochemical detection owing to their unique physicochemical properties. In the context of biochemical detection, the main advantages of AuNPs are their easy functionalization, enhanced signal strength, good biocompatibility, and high sensitivity^23^. Research on the combination of CMUTs and Au nanoparticles for biosensing, all of which are based on device surface modification, is relatively rare^24^.

In this study, we first devised a detection method for cancer biomarkers based on the Rayleigh scattering of CMUT sound waves by AuNPs, which results in attenuation of the received voltage. A pair of CMUTs was fixed on opposite sides of the acrylic partition to transmit and receive ultrasonic waves. Polydimethylsiloxane (PDMS) was attached to an acrylic substrate to form an insertable substrate for modifying the primary antibody. When the antigen was present in the test mixture, the secondary antibody@AuNP-2 and the antigen simultaneously bound to the insertable modified substrate. The nanoparticles induced Rayleigh scattering of sound waves, resulting in attenuation of the received sound energy and a change in the received sound pressure, thereby revealing the presence and concentration of the antigen. The expression of the acoustic pressure after the emitted acoustic pressure was Rayleigh-scattered by multiple nanoparticles provides a theoretical basis for biochemical detection based on the combination of CMUTs and nanoparticles. Initially, the relative change in the receiving voltage was proposed as the sensing response. A biochemical experiment was subsequently conducted in the detection chamber using a homemade CMUT chip, and the influences of the nanoparticle material and particle size on the CMUT receiving voltage were studied. Finally, the antigen was detected via AuNPs modified with antibodies. This detection method can be highly integrated in the future for rapid multitarget detection. Therefore, by integrating advanced sensing technology, automated processing, and data analysis tools, detecting multiple targets in a short time will be possible. This highly integrated detection method will significantly improve detection efficiency and accuracy and will be widely used in various fields, such as environmental monitoring, food safety testing, and biomedical analysis.

## Results and discussion

### Detection principle

**Fig. 1:**
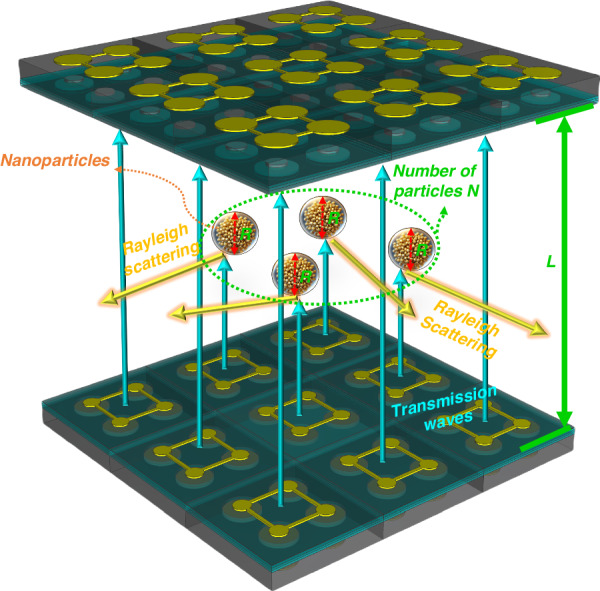
Ultrasonic Rayleigh scattering mechanism caused by nanoparticles

Rayleigh scattering refers to the phenomenon that occurs when the size of the particles is much smaller than the wavelength of the incident wave. Specifically, when the wavelength $\lambda$ is much larger than the particle diameter $a$ (typically $a\ll \lambda$), the particle can be considered an electric dipole. Under the influence of an incident wave, the particle becomes polarized, resulting in scattering. In this case, the scattering characteristics are mainly determined by the electric dipole moment of the particle and are independent of the particle shape and structure. The primary mechanism of Rayleigh scattering is elastic scattering by small particles. When the wavelength of sound waves is much larger than the particle size, the sound wave does not undergo significant reflection or diffraction upon encountering particles; instead, it causes slight compression and rarefaction around the particles. These variations result in small changes in the density of the medium around the particles, leading to wave scattering^25,26^.

As shown in Fig. 1, considering the cumulative effect of multiple scattering events, the wave intensity continuously decreases as it propagates. By using the Beer‒Lambert law, we can quantify this decrease and predict the remaining intensity of the sound wave after traveling a certain distance in the medium. *L* is the propagation distance between two transducers, *N* is the number of nanoparticles, and *R* is the particle size of nanoparticles.

### Attenuation model

As the sound wave propagates through the medium, the intensity decreases due to scattering by particles. The rate of change in intensity *I* with respect to a distance *L* is proportional to the intensity itself and the number density of scatterers^27^:

1 $$\frac{dI}{dL}=-N{\sigma }_{S}I$$

This differential equation states that the change in intensity over a small distance $dI$ is proportional to the product of the number of particles per unit volume *n*, the scattering cross-section ${\sigma }_{S}$, and the intensity *I*. To find the intensity after traveling a distance *L*, we integrate the above differential equation:

2 $${\int }_{\!\!{I}_{0}}^{I(L)}\frac{dI}{I}=-{\int }_{\!\!0}^{L}N{\sigma }_{S}dL$$

This equation simplifies to

3 $$In\left(\frac{I(L)}{{I}_{0}}\right)=-N{\sigma }_{s}L$$

Therefore, the intensity *I*(*L*) after traveling a distance *L* through the medium is

4 $$I(L)={I}_{0}{e}^{-N{\sigma }_{s}L}$$

To calculate the total scattering cross-section ${\sigma }_{S}$ of a single particle by integrating the differential scattering cross-section over the cross-sectional area, the differential scattering cross-section describes the angular distribution of the scattering intensity of a single particle, providing the intensity of the scattered wave per unit solid angle as a function of the scattering angle. For Rayleigh scattering, the differential scattering cross-section function can be derived from the scattering amplitude function^28^.

The differential scattering cross-section for Rayleigh scattering is derived from the Poynting vector. Consider a small particle with radius *R* that is much smaller than the wavelength *λ* of the incident wave satisfying the Rayleigh scattering condition. The particle is assumed to be in a homogeneous medium, and an incident sound wave impinges on the particle, causing scattering^29^.

The Poynting vector represents the energy flux density of a wave^30^. For sound waves, the Poynting vector *S* can be expressed as:

5 $${S}_{SC}=\frac{1}{2}\mathrm{Re}({p}_{sc}{v}_{sc}^{\ast })$$

where ${p}_{sc}$ is the sound pressure, ${v}_{sc}$ is the particle velocity, Re denotes the real part, and * denotes the complex conjugate.

The particle velocity of the scattered wave is

6 $$v=\frac{1}{i\omega \rho }\nabla p$$

where $\rho$ is the density of the medium, $\omega$ is the angular frequency, and $\nabla$ denotes the gradient operator.

To determine the pooling vector of the scattered wave, we need to find the sound pressure and particle velocity of the scattered wave. Under Rayleigh scattering conditions, the scattered wave can be expressed in terms of spherical harmonics expansion^31^:

7 $${p}_{sc}={B}_{1}{h}_{1}^{(1)}(kr){Y}_{10}(\theta ,\phi )$$

Substituting the expressions for the sound pressure and particle velocity of the scattered wave into the Poynting vector equation, we obtain

8 $${S}_{SC}=\frac{1}{2}\mathrm{Re}({B}_{1}{h}_{1}^{(1)}(kr){Y}_{10}(\theta ,\phi ).\frac{1}{i\omega \rho }\nabla ({B}_{1}^{\ast }{h}_{1}^{(1)\ast }(kr){Y}_{10}^{\ast }(\theta ,\phi )))$$

In the far-field region, the spherical Hankel function of the first kind can be approximated as

9 $${h}_{1}^{(1)}(kr)\approx -i\frac{{e}^{ikr}}{kr}$$

The spherical harmonic function ${Y}_{10}(\theta ,\phi )$ is expressed as

10 $${Y}_{10}(\theta ,\phi )=\sqrt{\frac{3}{4\pi }}\,\cos \theta$$

Therefore, the sound pressure of the scattered wave in the far field can be expressed as

11 $${p}_{sc}\approx -i{B}_{1}\frac{{e}^{ikr}}{kr}{Y}_{10}(\theta ,\phi )\approx -i{B}_{1}\frac{{e}^{ikr}}{kr}\sqrt{\frac{3}{4\pi }}\,\cos \theta$$

The particle velocity of the scattered wave is

12 $${v}_{sc}=\frac{1}{i\omega \rho }\nabla ({B}_{1}\frac{{e}^{ikr}}{kr}{Y}_{10}(\theta ,\phi ))$$

Substituting the Poynting vectors of the scattered and incident waves into the definition of the differential scattering cross-section, we obtain

13 $$\frac{d{\sigma }_{s}}{d\varOmega }=\frac{|{S}_{SC}|}{|{S}_{0}|}=\left|\frac{{p}_{sc}}{{p}_{0}}\right|^{2}$$

For Rayleigh scattering, the scattering amplitude is related to the particle radius $R$ and the wavelength $\lambda$. Specifically, ${B}_{1}$ can be expressed as

14 $${B}_{1}={p}_{0}{\left(\frac{R}{\lambda }\right)}^{3}\alpha$$

where $\alpha$ is the acoustic polarizability: $\alpha =\frac{4}{3}\pi {R}^{3}(\frac{{\rho }_{p}-{\rho }_{0}}{{\rho }_{p}+2{\rho }_{0}})$^32^, where ${\rho }_{p}$ is the density of the particle and ${\rho }_{0}$ is the density of the medium.

To obtain the full angular dependence, considering the dipole radiation pattern of Rayleigh scattering, the angular dependence term is $\frac{1+{\cos }^{2}\theta }{2}$.

Therefore, the differential scattering cross-section is

15 $$\frac{d{\sigma }_{s}}{d\varOmega }={\left(\frac{R}{\lambda }\right)}^{6}\left|\frac{4}{3}\pi {R}^{3}\left(\frac{{\rho }_{p}-{\rho }_{0}}{{\rho }_{p}+2{\rho }_{0}}\right)\right|^{2}{\left(\frac{1+{\cos }^{2}\theta }{2}\right)}^{2}$$

The total scattering cross-section is calculated by integrating the differential scattering cross-section over all cross-sections^33^:

16 $${\sigma }_{s}={\int }_{\!\!0}^{2\pi }{\int }_{\!\!0}^{\pi }\frac{d\sigma }{d\varOmega }\sin \theta d\theta d\phi =\left.{\left(\frac{R}{\lambda }\right)}^{6}\right|\frac{4}{3}\pi {R}^{3}\left.\left(\frac{{\rho }_{p}-{\rho }_{0}}{{\rho }_{p}+2{\rho }_{0}}\right)\right|^{2}{\left(\frac{1+{\cos }^{2}\theta }{2}\right)}^{2}\frac{28\pi }{15}$$

From Eqs. (4) and (15), we can obtain

17 $$I={I}_{0}{e}^{-LN\left.{\left(\frac{R}{\lambda }\right)}^{6}\right|\frac{4}{3}\pi {R}^{3}\left.\left(\frac{{\rho }_{p}-{\rho }_{0}}{{\rho }_{p}+2{\rho }_{0}}\right)\right|^{2}{\left(\frac{1+{\cos }^{2}\theta }{2}\right)}^{2}\frac{28\pi }{15}}$$

The intensity $I$ is related to the sound pressure ${p}^{\prime}$ as $I=\frac{{P}^{\text{'}2}}{2{\rho }_{0}{c}_{0}}$. The final sound pressure after multiple scattering events can be obtained via the following formula:

18 $${p}^{{\prime}}={p}_{0}{e}^{-LN{\left(\frac{R}{\lambda }\right)}^{6}\left|\frac{4}{3}\pi {R}^{3}\left(\frac{{\rho }_{p}-{\rho }_{0}}{{\rho }_{p}+2{\rho }_{0}}\right)\right|^{2}{\left(\frac{1+{\cos }^{2}\theta }{2}\right)}^{2}\frac{14\pi }{15}}$$

### Design and characterization of the CMUT chip

**Fig. 2:**
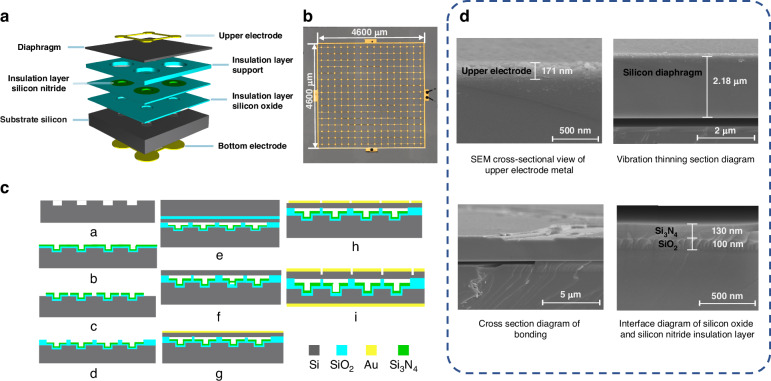
CMUT design and characterization. **a** Structural diagram. **b** Surface morphology diagram of the device. **c** Process flow diagram. **d** SEM image of the cross-section of the unit device structure

To perform biochemical measurements, the CMUT chip was first designed and processed, and its structural schematic is shown in Fig. 2a. The CMUT chip consists of an Au top electrode, a Si membrane, SiO_2_ and silicon nitride insulating layers, a Si substrate, and a Au bottom electrode. Figure 2b shows an optical microscope image of the CMUT chip, which consists of 16 × 16 elements with a total size of 4.9 × 4.9 mm.

The Rayleigh scattering of sound waves emitted by CMUTs induced by AuNPs is used for the detection of insertable cancer biomarkers. To meet the requirements of biomarker detection applications, high-frequency CMUTs with a sound wavelength much larger than the diameter of the nanoparticles were designed. Considering the device performance and fabrication processes, a membrane with a diameter of 140 μm and a thickness of 2.5 μm was designed, with a cavity size of 0.8 μm allowing for a bias voltage of 180 V and a natural frequency of 1.85 MHz. The top electrode (Ti/Au) had a diameter of 70 μm and a thickness of 170 nm, covering the vibrating area of the Si membrane. To prevent short circuits between the upper and lower electrodes, SiO_2_ and Si_3_N_4_ insulation layers with thicknesses of 0.1 μm and 0.12 μm, respectively, were laid at the bottom of the cavity. Si with a resistivity of <15–25 Ω·cm and a thickness of 425 μm was used as the device substrate, with the back of the substrate Si having a 0.2 μm-thick Au layer serving as the bottom electrode bonded to the printed circuit board. The steps of the fabrication process are shown in Fig. 2c. Lightly doped Si wafers were chosen as the substrates. After RCA standard cleaning, the wafers were patterned via photolithography and uniformly etched via deep reactive ion etching (DRIE) to form cavities (Step a). Insulating layers of SiO_2_ and silicon nitride were deposited via thermal oxidation (dry thermal oxidation) and low-pressure chemical vapor deposition. Silicon nitride not only provides insulation protection for the device but also facilitates secondary thermal oxidation to form cavities (Step b). The Si_3_N_4_ insulation layer was dry-etched, and the SiO_2_ film was wet-etched with a buffered oxide etchant (BOE) solution (Step c). Secondary thermal oxidation (a combination of dry thermal oxidation and wet thermal oxidation) was performed to form SiO_2_ insulating support pillars (Step d). Using a high-temperature (400 °C) wafer bonding process, the structural Si wafer was bonded to a silicon-on-insulator (SOI) wafer (Step e). The handle layer of the SOI was removed via mechanical thinning and DRIE dry etching, followed by wet etching of the SiO_2_ insulation layer with a BOE solution (Step f). The top electrode (Ti/Au) was deposited via physical vapor deposition magnetron sputtering (Step g), followed by photolithographic patterning and physical ion beam etching (Step h). Finally, the Si substrate was thinned to 0.2 μm, and the metal bottom electrode (Ti/Au) was deposited (Step i). Through these steps, the fabrication of a high-frequency CMUT was completed. The electromechanical coupling coefficient, transmission performance, and reception performance of the CMUT were subsequently tested. Figure 2d shows a cross-sectional view of the device, which was obtained by scanning electron microscopy (SEM), revealing a membrane thickness of 2.17 μm, a top electrode thickness of 0.17 μm, and actual parameters of the 0.1 μm SiO_2_ and 0.13 μm Si_3_N_4_ insulation layers. Additionally, to ensure the quality of the Si–SiO_2_ bonding, the micropore spacing was reduced to 140 μm, maximizing the fill density and improving the performance of the CMUT.

### Performance testing of the CMUT chip

The impedance phase‒frequency curve of the CMUT chip was measured via an impedance analyzer (E4990A. Agilent Technologies). This measurement helps determine the resonance frequency of the CMUT chip in air and assists in calculating the parameters for possible impedance matching components.

**Fig. 3:**
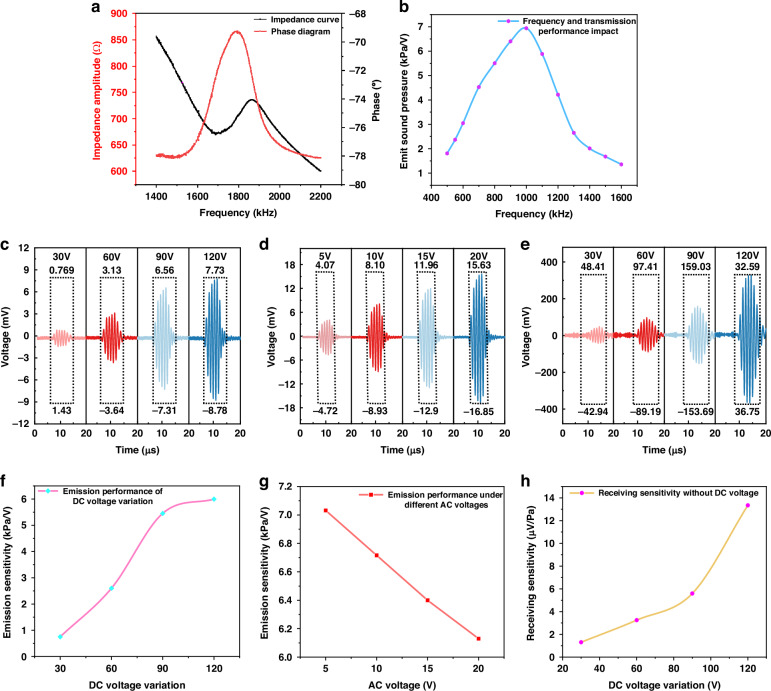
Performance testing of the CMUT chip. **a** CMUT impedance curve. **b** Variation of the emission sensitivity of CMUT chips with frequency. **c**–**f** Emission performance of the CMUT chip under different DC voltages. **d**–**g** Transmitting performance of the CMUT chip under different AC voltages. **e**–**h** CMUT chip receiving performance under different DC voltages

Figure 3a shows the CMUT chip. Under a voltage bias of 120 V, the resonant frequency is 1.68 MHz, and the antiresonant frequency is 1.87 MHz. The electromechanical coupling coefficient of the CMUT was calculated to be 19.2%.

The experimental tests were conducted via a commercial needle hydrophone (NH2000, PA, USA). The results showed that our CMUT chip had a bandwidth of 110% (–6 dB) at a distance of 10 mm in deionized water (this distance corresponds to the strongest emission sound pressure under the same bias and AC voltage) (Fig. 3b). Figure 3c, d, f, and g show the changes in the emission performance along the axis of the CMUT chip under different DC or AC voltages. The test results indicate that when a fixed AC voltage of 10 V is applied, the emission sensitivity increases with increasing DC voltage, reaching a maximum emission sensitivity of 6 kPa/V at a DC bias of 120 V. When a fixed DC voltage of 120 V is applied, the emission sensitivity decreases with increasing AC voltage. Figure 3e, h demonstrate that the reception sensitivity of the CMUT increases with increasing DC bias voltage, reaching a maximum reception sensitivity of 13.3 μV/Pa.

To obtain the optimal voltage response for biochemical detection, two CMUT chips were placed on either side of a 10 mm-thick acrylic spacer. One chip was driven by a 10 V AC voltage and a 120 V bias voltage, whereas the other CMUT chip was used as a receiver with a 120 V DC bias voltage. The experiments indicated that the CMUT chip produced the highest output voltage at a resonant frequency of 1 MHz during tests in deionized water. Therefore, in the subsequent biochemical measurements, the operating frequency of the CMUT chip was set to 1 MHz.

### Experimental procedures

**Fig. 4:**
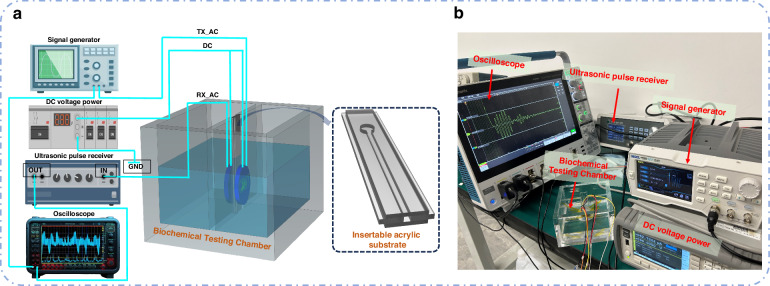
Experimental system. **a** Schematic of the measurement system. **b** Digital image of the measurement system

To demonstrate the feasibility of the CMUT working principle combined with nanoparticle biochemical detection, we designed a preliminary experiment to study the effects of nanoparticle changes on the CMUT receiving voltage. Figure 4a shows the ultrasonic biochemical detection system consisting of a signal generator, DC voltage power supply, ultrasonic pulse receiver, oscilloscope, and biochemical testing chamber. The signal generator (DG-1032Z, Rigol) and DC voltage power supply (2612 A, KEITHLEY) provided AC and DC excitation voltages for the CMUT, respectively. An ultrasonic pulse receiver was used to filter and amplify the received signal, and an oscilloscope (MSO44, Tektronix) was employed to display and record the received signal. A schematic of the detection chamber is shown, in which the insertable acrylic substrate, acrylic solution tank, and CMUT constitute the detection chamber. A CMUT array (16×16 circular units) of the same size as the prepared array was used as the ultrasonic transmitter and receiver. The distance between the transmitting and receiving ultrasonic transducers was 10 mm. The insertable acrylic substrate was located between the two CMUTs and consisted of a three-layer structure for injecting the trace nanoparticle solutions. AuNPs and Ag nanoparticles (AgNPs) were synthesized via the method described in Supplementary Information. Experimental tests were performed with a fixed solution volume and position for water, AuNP solution, and the mixed nanoparticle solution on an acrylic substrate. The detection chamber was composed of acrylic plates, and deionized water was injected into it. The ambient temperature was maintained at 26 °C, and the tests were conducted under a 120 V DC bias and a 10 V AC voltage, with an excitation frequency of 1 MHz and five pulses. The ultrasonic emission period was set to 1 ms. To ensure accuracy, continuous ultrasonic emission was used, and the average of multiple signals collected by the oscilloscope was extracted. Figure 4b shows a digital picture of the entire experimental setup.

### Verification of the biochemical detection feasibility

**Fig. 5:**
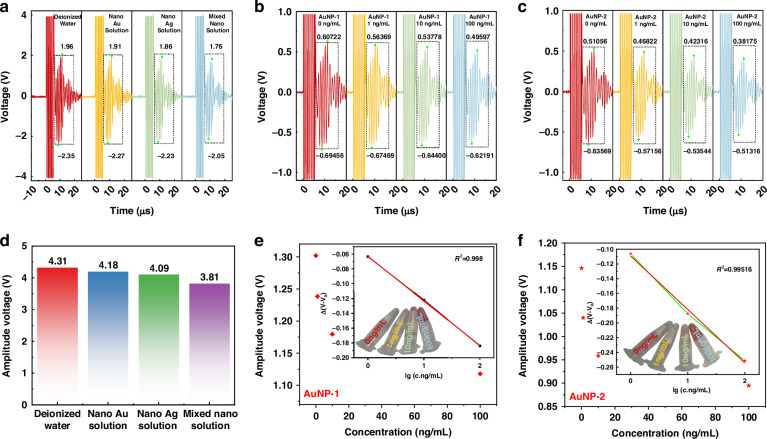
Nanoparticle test on CMUT receiving performance. **a**, **b** Effects of nanoparticles of different materials on the CMUT receiving waveform and amplitude. **b**, **e** Effects of different concentrations of AuNP-1 on the receiving waveform and amplitude voltage. **c**, **f** Effects of different concentrations of AuNP-2 on the receiving waveform and amplitude voltage

The characterization of the nanoparticles in Supplementary Information shows that AuNPs-1, AuNPs-2, and AgNPs have diameters of ~12.49 ± 1.54, 54.41 ± 5.49, and 60.34 ± 19.56 nm, respectively. The experimental results of the nanoparticle solution test are presented in Fig. 5a and d. When nanoparticles are present in an aqueous solution, the Rayleigh scattering effect of the nanoparticles on sound waves reduces the received voltage signal compared with the voltage signal of a pure aqueous solution. For different materials with Au and Ag nanoparticles at the same concentration (2.5 nM), the signal received by the AgNPs is weaker in the main band, which is related to the particle size and density of the Ag nanoparticles, and the acoustic wave attenuates faster in the solution containing AgNPs. As shown in Fig. 5b, c, e, and f, AuNPs-1 and AuNPs-2 exhibit stronger Rayleigh scattering with increasing concentration, resulting in weaker received voltage signals. At the same concentration, the main band of the solution of AuNPs-2 received weaker signals than did the solution of AuNPs-1. This experimental combination is consistent with the theory of ultrasonic Rayleigh scattering by the nanoparticles mentioned above. The above calculation theory demonstrates that as the particle size increases, the Rayleigh scattering becomes stronger. Additionally, as the concentration increases, the scattering intensity increases with N, and the received voltage becomes weaker.

### CA19-9 biochemical testing of cancer markers

**Fig. 6:**
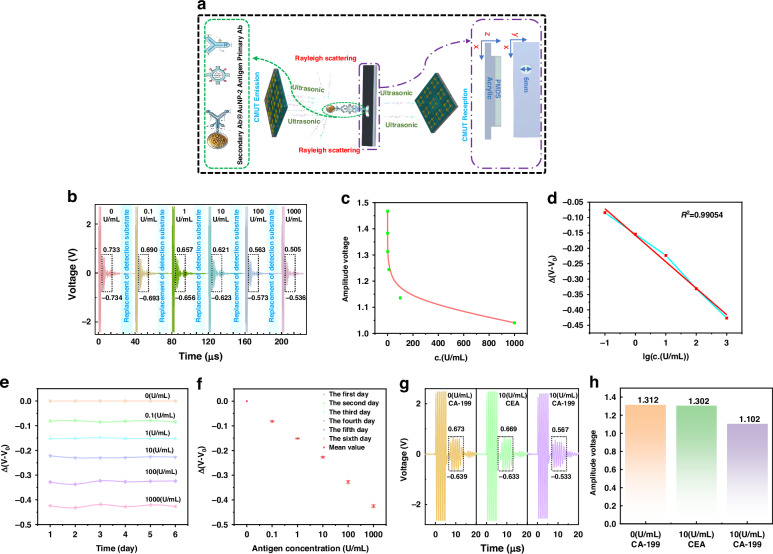
CA19-9 biochemical testing of cancer markers. **a** CA19-9 antigen detection principle diagram. **b** Waveform of the CMUT receiving signal under different concentrations of CA19-9 antigen. **c**, **d** Relative changes in the receiving voltage under different antigen concentrations. **e**, **f** Results of six consecutive days of repeatability testing of different antigen concentrations. **g**, **h** Effects of adding antigens CAE and CA19-9 on the CMUT receiving waveform and amplitude voltage

Next, we used the detection system and biochemical testing chamber (Fig. 4a) to detect the CA-199 antigen marker. The ambient temperature was maintained at 26 °C, and the tests were conducted under a 120 V DC bias and a 10 V AC voltage, with an excitation frequency of 1 MHz and five pulses. The temperature of the solution in the biochemical testing chamber and the surrounding environment remained constant and consistent, and no bubbles were generated. Figure 6a shows the principle of CA19-9 antigen detection. The inserted antibody-modified substrate was located between two CMUTs and consisted of a double-layered structure of PDMS and an acrylic plate. PDMS was used to modify the primary antibodies, capture antigens and capture secondary antibody@AuNP-2 in the detection solution. The PDMS modification process for the primary antibody is shown in Fig. S2 of Supplementary Information. When antigens are present in the solution, the emitted sound waves encounter the AuNP-2-modified secondary antibody and undergo Rayleigh scattering, resulting in a reduced received sound wave intensity. Additionally, the received voltage weakens as the concentration increases. Figure S3 shows the process of CA19-9 antigen testing.

The measurement results in Fig. 6b, c show that the detection of the CA19-9 antigen marker is rapid, with a detection time of < 250 μs, and the CMUT receiving voltage decreases to varying degrees with increasing CA19-9 antigen concentration. The detection limit reaches 0.1 (U/mL), whereas the normal value of clinically relevant carbohydrate antigen (CA19-9) is < 37.00 (U/mL)^34^; therefore, our method meets practical needs. Figure 6d shows the relationship between the concentration of CA19-9 and the CMUT receiving voltage. The *R*^*2*^ value is 0.991, and the sensitivity reaches 80 mV per decade. Figure 6e, f show the experimental results of repeated antigen testing. Slight fluctuations exist in the voltage received during the repeated tests over six consecutive days. The standard deviations of the received voltages for CA-199 from 0 to 1000 (U/mL) are 0, 0.00271, 0.0016, 0.00308, 0.00509, and 0.00479 V and are all < 3% of the average received voltage with good repeatability.

Selectivity refers to the ability of a biochemical assay to produce positive results from specific interactions. Figure 6g, h confirm the specificity of the CA19-9 assay, in which the CAE antigen is used for interference testing. The addition of CA19-9 results in significant voltage changes. In contrast, when the CAE is added, the average change in the signal is negligible. The test results demonstrate that the detection system is not affected by interfering substances.

Compared with traditional methods of CA19-9 detection, our system demonstrates better performance in terms of the detection range and limit of detection; the results are summarized in Table 1.

**Table 1:** Comparison of the state-of-the-art analytical performance of different biosensors for CA19-9 detection

Test method	Method	Linear range (U/mL)	Detection limit (U/mL)	Ref
**EIS**	Zn–Co–S/graphene	6.3–300	0.82	^35^
**EIS**	Polythionine-Au composites (AuNPs@PThi)	6.5–520	0.26	^36^
**DPV**	Au Ag HNCs/GCE	1–30	0.28	^37^
**EIS**	GO	0.3–100	0.12	^38^
**PM-IRRAS**	PEI-MWCNT	0.5–60	0.35	^39^
**SPR**	GO-COOH	10–1000	10	^40^
**This work**	CMUT-Nano Au	0.1–1000	0.1	

## Conclusion

We demonstrated a technique for detecting cancer markers using CMUTs in combination with nanoparticles. A pair of CMUTs was directly clamped on both sides of the biochemical detection chamber for the emission and reception of the ultrasound waves. The presence and concentration of antigens were measured via the scattering of acoustic waves caused by the antibody-modified nanoparticles, resulting in the attenuation of the acoustic energy and a change in the received voltage. Theoretical expressions for the received acoustic pressure and size and concentration of the nanoparticles were established and verified through nanoparticle experiments. We verified that the relative voltage change decreased with increasing nanoparticle size and decreased with increasing nanoparticle concentration. After verifying that the voltage received by the CMUT was affected by the size, material, and concentration of the nanoparticles, it was combined with cancer antibodies for the detection of CA19-9 cancer antigens. The results showed that the detection method had a low detection limit (0.1 U/mL), good sensitivity (80 mV per decade), and good linearity (*R*^2^ = 0.991). After optimization, this biochemical marker detection technique can be utilized for detecting biological samples in whole blood and serum. These features suggest that our method could become a competitive approach for use in medical monitoring and clinical diagnosis.

## Supplementary information

Supplemental Material
