# Supplementary material for: Biomarker detection based on nanoparticle-induced ultrasonic Rayleigh scattering
Source: Microsyst Nanoeng. 2024 Dec 5;10:182. doi: 10.1038/s41378-024-00808-z (PMC11618333; doi:10.1038/s41378-024-00808-z)
Supplement: Supplementary file 1 — Supplemental Material [file 41378_2024_808_MOESM1_ESM.docx]

**Supplementary Information**

**Biomarker Detection Based on Nanoparticle-Induced Ultrasonic Rayleigh Scattering**

Wangyang Zhang, Chaoshan Zhao, Haoliang Jia, Tao Liu, Jiaqian Yang, Pengfan Wu, Xiaojing Mu*

**Key Laboratory of Optoelectronic Technology and Systems, Ministry of Education and International Research and Development Center of Micro-Nano Systems and New Materials Technology, Chongqing University, Chongqing 400044, China**

**Supplementary files include:**

**Characterization of the NPs**

**Supplementary Figures Sl to S3**

| Characterization of the NPs | TEM characterization of AuNPs-1, AuNPs-2, and AgNP |
| --- | --- |
| Figures Sl | UV absorption spectra of secondary antibody@AuNPs-2 and unmodified AuNPs-2. |
| Figures S2 | CA19-9 antibody-modified PDMS process. |
| Figures S3 | CA19-9 antigen testing process |

**General Procedures.**

All reactions were performed in dry glassware with strict air and moisture exclusion using standard Schlenk or cannulation techniques. All chemicals were purchased from Sigma–Aldrich or Acros Chemicals.

The UV‒visible spectra were obtained using a Lambda 950 UV/VIS/NIR spectrometer at a wavelength of nanometers. The AuNPs were characterized via transmission electron microscopy (TEM) with a Tecnai G2 F20 U-TWIN transmission electron microscope. All the TEM images were obtained at 200 keV. The size distributions of the AuNPs and AgNPs were analyzed via ImageJ software. A few drops of the nanoparticle solution were placed on a C-coated 300 mesh Cu TEM grid, excess solvent was removed via filter paper, and each sample was allowed to dry under ambient conditions.

**Synthetic Procedures**

Synthesis of AuNPs-1

Using the standard citrate reduction method, AuNPs-1 were prepared as reported previously^[1]^. Briefly, 0.5 mL of HAuCl_4_•3H_2_O solution (1%, w/v) in 50 mL of Milli-Q water was heated to boiling, and 1.5 mL of sodium citrate solution (1%, w/v) was quickly added to the boiling solution with vigorous stirring. After the color change was completed within 5 min, the mixture was boiled for another 15–30 min and then allowed to cool to room temperature with stirring. Finally, AuNPs-1 were obtained, and the resulting nanoparticles were used as the seed solution to synthesize larger AuNPs measuring 54 nm.

Synthesis of AuNPs-2

The AuNPs-2 were synthesized via a seed growth method^[2]^. Generally, the AuNPs-1 seed mixture was mixed with 2.44 mL of HAuCl_4_•3H_2_O solution (10 mmol/L), and the mixture was diluted to 150 mL with Milli-Q water. Then, 100 mL of ascorbic acid solution (0.4 mmol/L) was added to the stirred solution at a rate of 10 mL/min via a peristaltic pump. Finally, AuNPs-2 (purple) were synthesized.

Synthesis of AgNPs

Colloidal AgNPs were synthesized via the reduction of a 1 mM AgNO_3_ complex with a 1% trisodium citrate solution with continuous stirring and boiling. The samples were collected from the reaction mixture at time intervals of 4, 6, 8, and 10 min, with colors of pale yellow, yellow, brown, and gray, respectively. Furthermore, the size distributions of these particles were evaluated^[3]^.

**Characterization of the NPs**


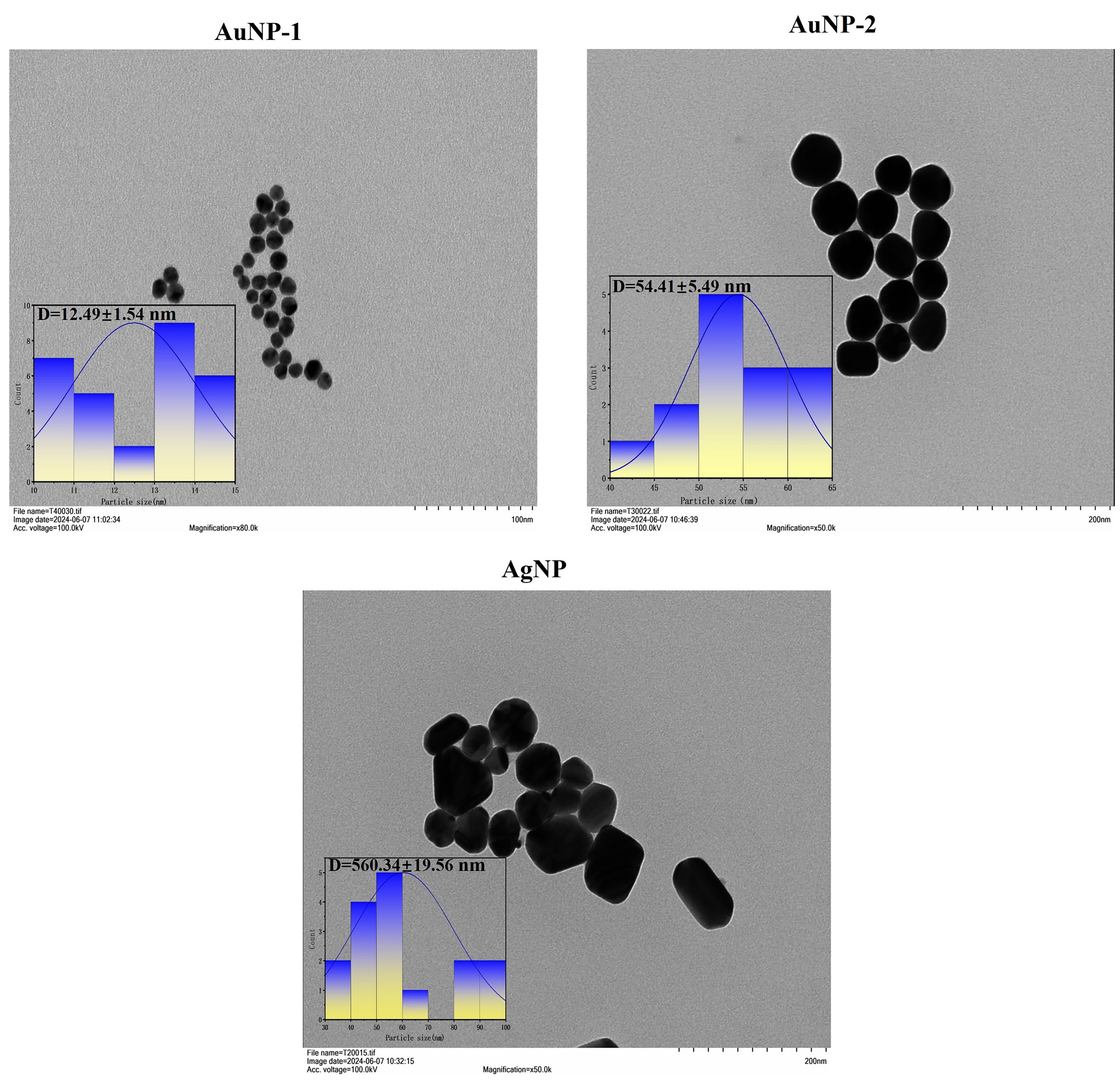


**Secondary Antibody@AuNP-2 Methods**

First, 1 mL of nanogold suspension was placed in a centrifuge tube, and 10 μL of 12% sodium dodecyl sulfate (to prevent aggregation) and 10 μL of 1 mM dithiobis succinimidyl propionate (DTSP, prepared with dimethylsulfoxide as the solvent) solution were added. The mixture was mixed thoroughly and left overnight at room temperature. Next, 100 μL of 10 μg/mL CA19-9 secondary antibody was added, and the mixture was incubated at room temperature for 2 h. DTSP was used as a crosslinking agent to bind the nanogold and CA19-9 secondary antibodies. The supernatant was then removed via centrifugation (centrifugation parameters: 12,000 rpm for 8 min)^[4]^.

Figure S1 shows the UV spectra of the AuNPs-2 modified with secondary antibodies and unmodified nanoparticles. The results indicate that the absorption peak of the nanoparticles modified with secondary antibodies increased, suggesting an increase in the size of the nanoparticles, which is consistent with actual observations.


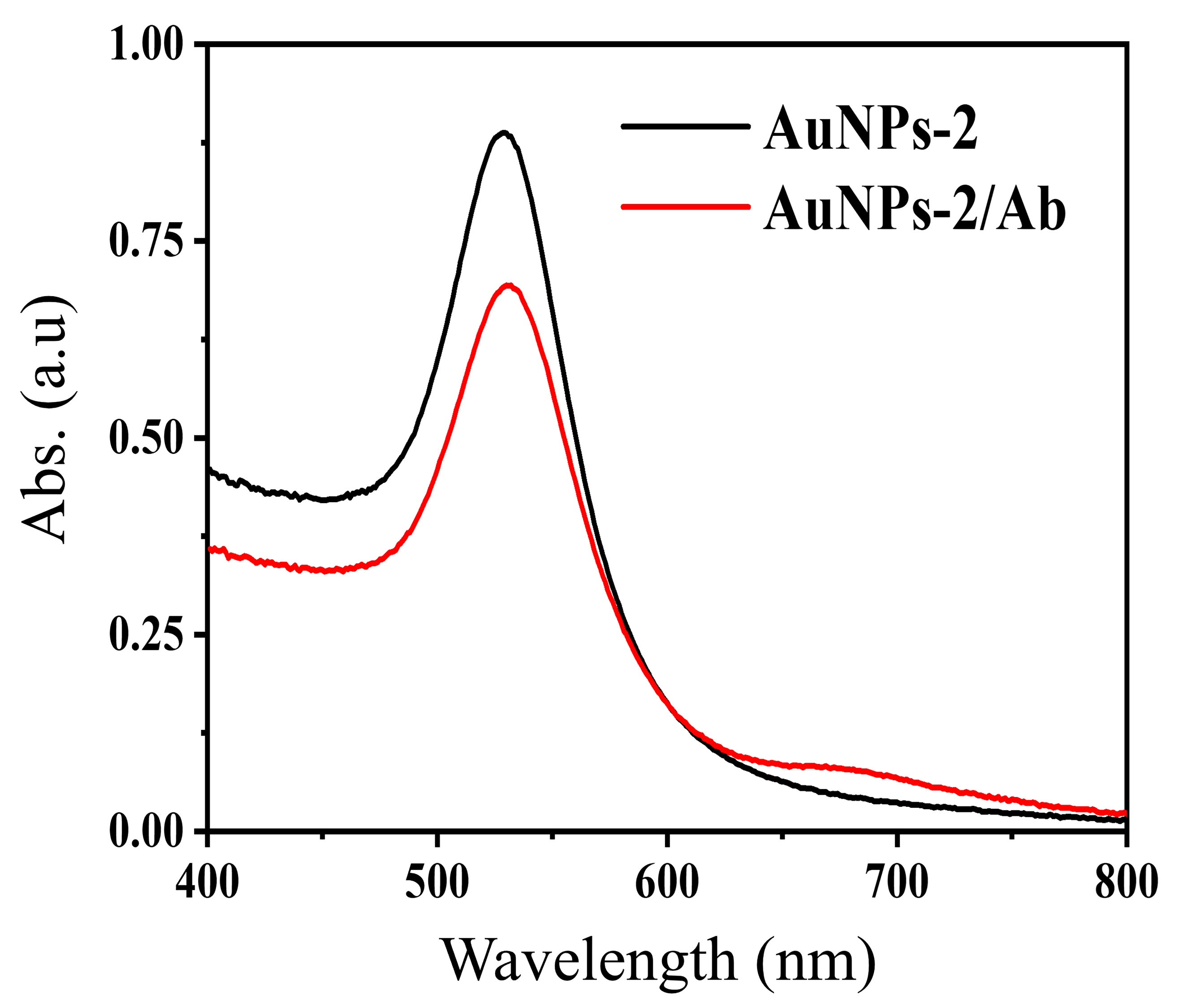


**Figure S1.** UV absorption spectra of secondary antibody@AuNPs-2 and unmodified AuNPs-2.

**CA19-9 Antibody-Modified PDMS Process**

Figure S2 shows the process of primary antibody modification of PDMS, which involved preparing the PDMS substrate, cleaning the PDMS surface, and performing activation via plasma treatment. The CA19-9 antibody solution was prepared at an appropriate concentration, placed in the PDMS, incubated at room temperature for 2 h, and gently shaken to ensure uniform coverage. The PDMS surface was gently rinsed with phosphate-buffered saline (PBS) to remove unbound antibodies and washed multiple times. The blocking buffer, bovine serum albumin (BSA), was added to the PDMS, which was subsequently incubated for 1 h and gently shaken. The PDMS surface was gently rinsed with PBS to remove the unbound blocking agent. Finally, the sample was dried naturally in a clean environment and stored at 4 °C under appropriate conditions until use^[5]^.


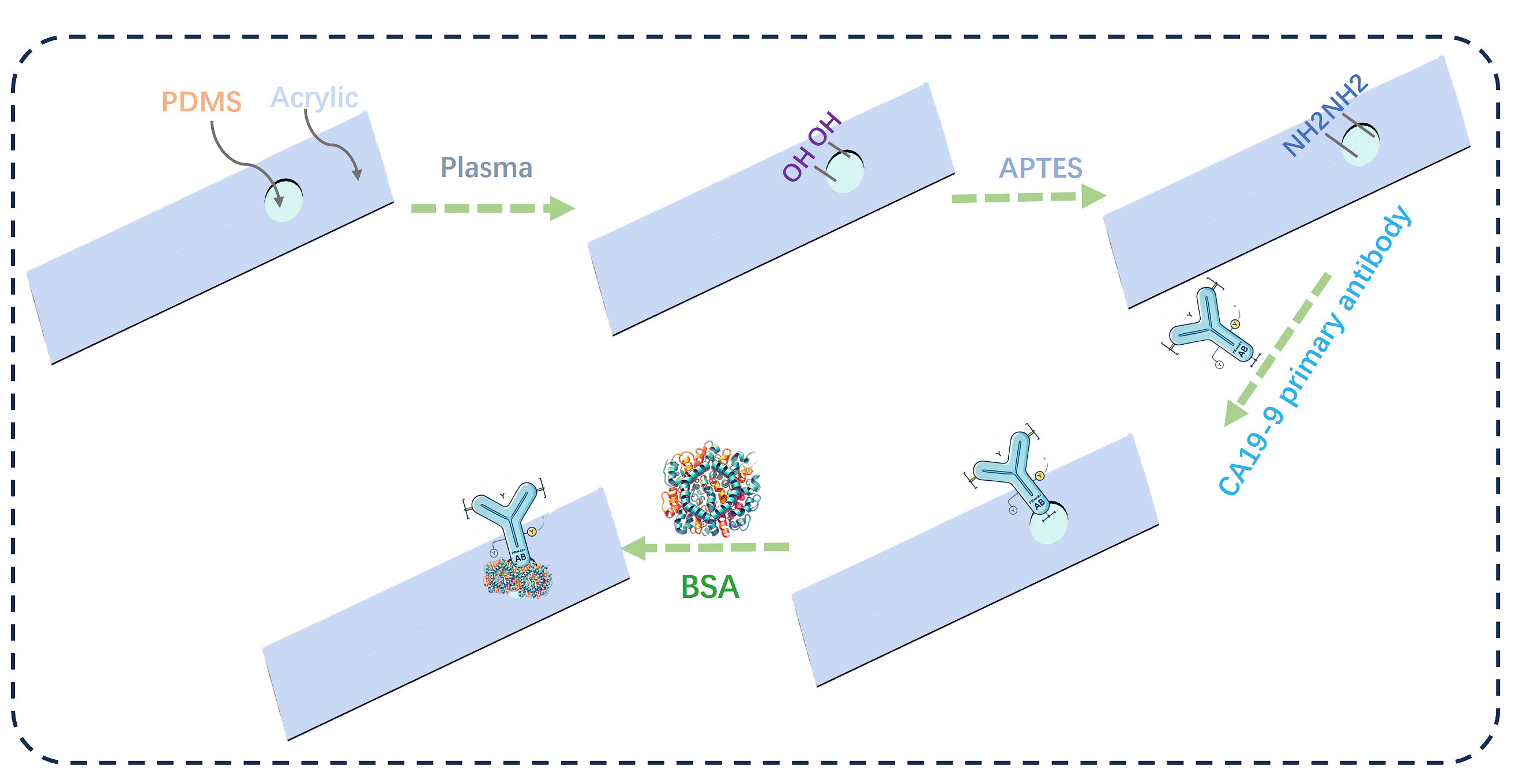


**Figure S2.** CA19-9 antibody-modified PDMS process.

**CA19-9 Antigen Testing Process**

Figure S3 shows the process of CA19-9 antigen testing. Figure S2 shows the process of primary antibody modification of PDMS. Next, antigens, including CA19-9 (0 U/mL), CA19-9 (0.1 U/mL), CA19-9 (1 U/mL), CA19-9 (10 U/mL), CA19-9 (100 U/mL), and CA19-9 (1000 U/mL), were added to 6 PDMS modified with the same concentration of primary antibody and incubated for 60 min. Then, secondary antibody@AuNP-2 was added to react with the CA19-9 antigen bound to the primary antibody for 60 min, followed by washing with PBST (0.05% Tween-20) for 5 min. The modified primary antibody PMDS substrate captured different concentrations of antigen and secondary antibody@AuNP-2, which can be inserted into the biochemical testing chamber in sequence for direct detection.


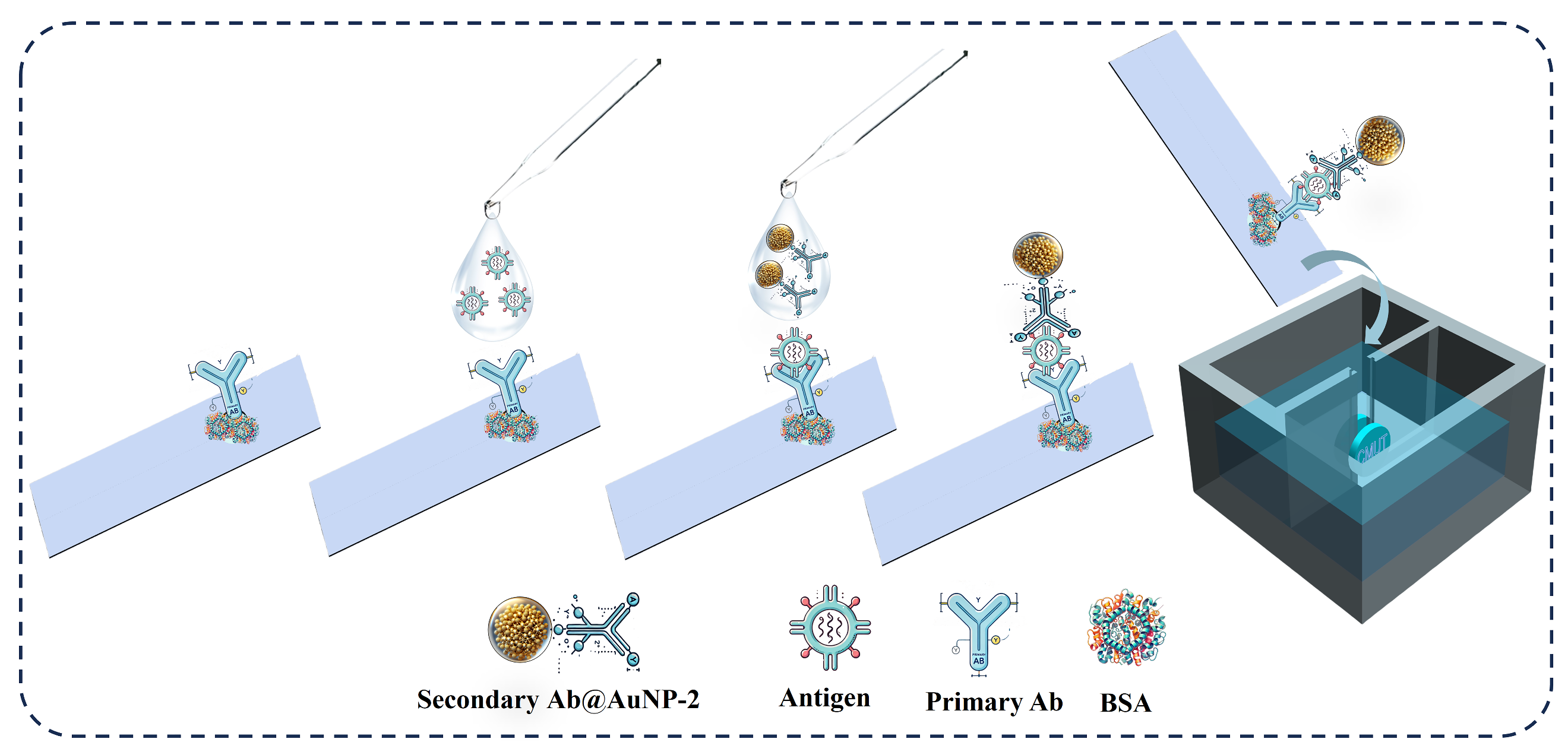


**Figure S3.** CA19-9 antigen testing process

**Reference**

1. Frens G. Controlled nucleation for regulation of particle-size in monodisperse gold suspensions. Nature-Physical Sci 1973;241:20–2.
2. Jana NR, Gearheart L, Murphy CJ. Evidence for seed-mediated nucleation in the chemical reduction of gold salts to gold nanoparticles. Chem Mater 2001;13:2313–22.
3. Pacios R, Marcilla R, Pozo-Gonzalo C, et al. Combined electrochromic and plasmonic optical responses in conducting polymer/metal nanoparticle films[J]. Journal of nanoscience and nanotechnology, 2007, 7(8): 2938-2941.
4. Ciaurriz, Paula, et al. "Comparison of four functionalization methods of gold nanoparticles for enhancing the enzyme-linked immunosorbent assay (ELISA)." Beilstein journal of nanotechnology 8.1 (2017): 244-253.
5. Huang, Chenghong, et al. "Label-free multiplex immunoassay of AFP, CEA and CA19-9 by integrated microfluidic biosensor based on imaging ellipsometry." Integrated Ferroelectrics 171.1 (2016): 59-69.
